# Meteorological Observations for July, 1857

**Published:** 1857-08

**Authors:** J. G. Westmoreland


### METEOROLOGICAL OBSERVATIONS FOR JULY, 1857, AT ATLANTA, GA.

| JULY. | THERMOMETER. | | | BAROMETER. | | | WIND. | REMARKS. |
|---|---|---|---|---|---|---|---|---|
| | 7 A. M. | 2 P. M. | 7 P. M. | 7 A. M. | 2 P. M. | 7 P. M. | | |
| 1 | 64 | 84 | 66 | 29.85 | 29.93 | 29.90 | S. W. | Hazy. |
| 2 | 56 | 78 | 62 | 27.83 | 29.93 | 29.85 | N. W. | Fair. |
| 3 | 58 | 80 | 60 | 27.95 | 29.95 | 29.90 | W. | Hazy. |
| 4 | 58 | 68 | 60 | 29.93 | 29.95 | 29.95 | E. | Cloudy—Rain ⅛. |
| 5 | 56 | 68 | 64 | 30.05 | 30.12 | 30.10 | N. W. | Cloudy—Drizzly. |
| 6 | 60 | 84 | 78 | 30.15 | 30.25 | 30.20 | E. | Fair. |
| 7 | 62 | 88 | 78 | 30.15 | 30.20 | 30.15 | N. | Fair. |
| 8 | 66 | 86 | 80 | 30.10 | 30.15 | 30.10 | E. | Fair. |
| 9 | 66 | 84 | 75 | 30.10 | 30.16 | 30.15 | W. | Fair. |
| 10 | 64 | 86 | 78 | 30.15 | 30.26 | 30.20 | E. | Fair. |
| 11 | 80 | 88 | 74 | 30.25 | 30.27 | 30.20 | W. | Fair. |
| 12 | 78 | 92 | 90 | 39.25 | 30.22 | 30.12 | W. | Cloudy—Rain ⅛. |
| 13 | 72 | 88 | 68 | 30.15 | 30.15 | 30.07 | W. | Hazy. |
| 14 | 70 | 84 | 76 | 30.05 | 30.10 | 30.05 | W. | Fair. |
| 15 | 68 | 90 | 80 | 30. | 30.02 | 29.95 | E. | Hazy. |
| 16 | 70 | 80 | 80 | 29.92 | 29.95 | 29.95 | S. E. | Cloudy—Rain ⅙. |
| 17 | 68 | 86 | 84 | 29.98 | 30.05 | 30.03 | S. E. | Cloudy. |
| 18 | 74 | 92 | 84 | 30.10 | 30.07 | 30.05 | W. | Fair. |
| 19 | 78 | 92 | 92 | 30.05 | 30.07 | 30. | S. W. | Cloudy—Rain ⅛. |
| 20 | 68 | 82 | 78 | 30. | 29.92 | 29.92 | S. E. | Fair. |
| 21 | 68 | 78 | 78 | 29.90 | 29.92 | 29.92 | W. | Cloudy—Rain ⅛. |
| 22 | 70 | 82 | 72 | 29.92 | 30. | 29.95 | N. W. | Hazy. |
| 23 | 66 | 84 | 78 | 29.95 | 30. | 30. | N. W. | Fair. |
| 24 | 70 | 78 | 72 | 30. | 30.07 | 30.10 | S. | Cloudy—Rain 1½. |
| 25 | 70 | 82 | 72 | 30.12 | 30.15 | 30.20 | S. | Cloudy—Rain 1. |
| 26 | 70 | 80 | 74 | 30.20 | 30.20 | 30.20 | S. | Cloudy. |
| 27 | 76 | 78 | 72 | 30.25 | 30.22 | 30.20 | S. | Cloudy—Rain ⅝. |
| 28 | 70 | 86 | 80 | 30.20 | 30.20 | 30.15 | S. | Cloudy—Rain 3-16. |
| 29 | 70 | 80 | 76 | 30.20 | 30.15 | 30.15 | S. W. | Cloudy—Rain ¼. |
| 30 | 72 | 84 | 72 | 30.15 | 30.15 | 30.15 | S. | Cloudy—Rain ¼. |
| 31 | 74 | 78 | 74 | 30.15 | 30.10 | 30.07 | S. W. | Cloudy—Rain ¼. |

Furnished by

J. G. WESTMORELAND, M. D.

